# A Hierarchical CuO Nanowire@CoFe-Layered Double Hydroxide Nanosheet Array as a High-Efficiency Seawater Oxidation Electrocatalyst

**DOI:** 10.3390/molecules28155718

**Published:** 2023-07-28

**Authors:** Xiya Yang, Xun He, Lang He, Jie Chen, Longcheng Zhang, Qian Liu, Zhengwei Cai, Chaoxin Yang, Shengjun Sun, Dongdong Zheng, Asmaa Farouk, Mohamed S. Hamdy, Zhaogang Ren, Xuping Sun

**Affiliations:** 1 College of Environmental Science and Engineering, China West Normal University, Nanchong 637009, China; 2 Institute of Fundamental and Frontier Sciences, University of Electronic Science and Technology of China, Chengdu 610054, China; 3 Institute for Advanced Study, Chengdu University, Chengdu 610106, China; liuqian@cdu.edu.cn; 4 College of Chemistry, Chemical Engineering and Materials Science, Shandong Normal University, Jinan 250014, Chinayangcx_sdnu@163.com (C.Y.);; 5 Department of Chemistry, College of Science, King Khalid University, Abha 61413, Saudi Arabia; afahmad@kku.edu.sa (A.F.);

**Keywords:** hierarchical CuO@CoFe-LDH nanoarray, alkaline seawater electrolysis, oxygen evolution reaction

## Abstract

Seawater electrolysis has great potential to generate clean hydrogen energy, but it is a formidable challenge. In this study, we report CoFe-LDH nanosheet uniformly decorated on a CuO nanowire array on Cu foam (CuO@CoFe-LDH/CF) for seawater oxidation. Such CuO@CoFe-LDH/CF exhibits high oxygen evolution reaction electrocatalytic activity, demanding only an overpotential of 336 mV to generate a current density of 100 mA cm^−2^ in alkaline seawater. Moreover, it can operate continuously for at least 50 h without obvious activity attenuation.

## 1. Introduction

Hydrogen (H_2_) is extensively considered an ideal future carbon-neutral energy carrier with high energy density, and water electrolysis is a facile, cost-effective, and environmentally friendly approach to producing high-purity H_2_ [1,2,3,4,5,6]. However, considering the limited reserves and uneven distribution on earth of freshwater, direct freshwater electrolysis will be a non-negligible problem for large-scale H_2_ production in the future [7,8]. Seawater, as an abundant water resource on earth, is regarded as a candidate to substitute for freshwater feedstock. However, the practical applications of seawater electrolysis still face formidable challenges, including the slow reaction kinetics of oxygen evolution reaction (OER) and the competitive chlorine evolution reaction occurring on the anode [9,10,11,12,13,14,15]. Thus, it is highly necessary to explore high-active OER electrocatalysts in seawater.

Currently, IrO_2_ and RuO_2_ exhibit excellent electrocatalytic properties for OER, but their exorbitant price and scarcity severely limit their widespread commercial applications [16,17,18,19]. Among transition metal-based electrocatalysts, layered double hydroxides (LDHs), especially CoFe-LDH, have recently aroused interest on account of their cost-effectiveness, good intrinsic sites, and relative ease of preparation [20,21,22]. It is widely known that three-dimensional core–shell nanostructures have the advantages of durability and more active sites to contact with electrolytes, promoting catalytic activity [23,24,25]. Although several studies have reported seawater oxidation enabled by CoFe-LDH [26,27,28], CoFe LDH-based hierarchical core–shell structures for boosting seawater oxidation has not been explored so far.

Herein, we report the development of a CoFe-LDH nanosheet decorated CuO nanowire array on Cu foam (CuO@CoFe-LDH/CF) for seawater oxidation. Such CuO@CoFe-LDH/CF exhibits high OER electrocatalytic activity, demanding only overpotentials of 336 and 375 mV to generate large current densities (*j*) of 100 and 300 mA·cm^−2^ in alkaline seawater, respectively. Furthermore, CuO@CoFe-LDH/CF achieves stable continuous electrolysis for 50 h at a *j* of 200 mA·cm^−2^ in alkaline seawater without obvious activity attenuation.

## 2. Results and Discussion

**Figure 1:**
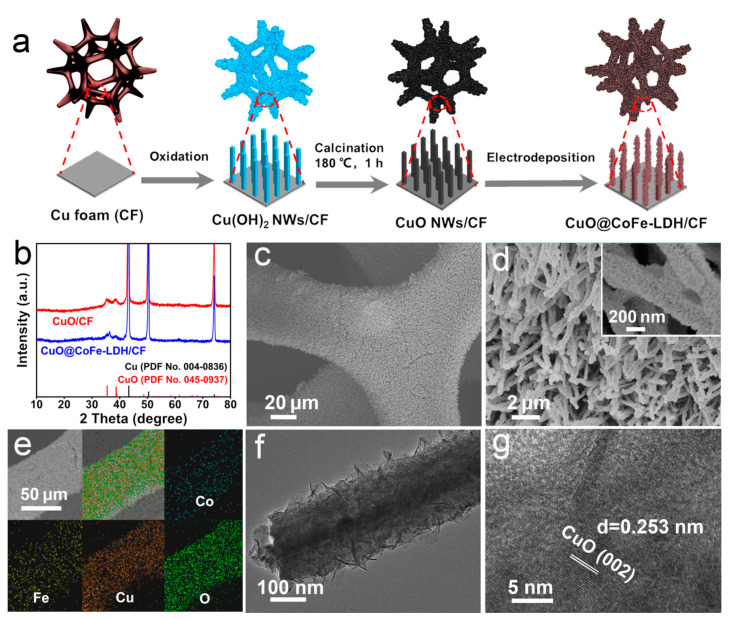
(**a**) Schematic fabrication process for CuO@CoFe-LDH/CF. (**b**) XRD patterns of CuO/CF and CuO@CoFe-LDH/CF. (**c**) Low- and (**d**) high-magnification SEM images of CuO@CoFe-LDH/CF. (**e**) SEM and corresponding EDX mapping images of Co, Fe, Cu, and O in CuO@CoFe-LDH/CF. (**f**) TEM image and (**g**) HRTEM image of CuO@CoFe-LDH.

A schematic diagram of the synthesis and optical photograph for the preparation of CuO@CoFe-LDH/CF is illustrated in Figure 1a and Figure S1, respectively. The X-ray diffraction (XRD) patterns of CuO/CF and CuO@CoFe-LDH/CF are presented in Figure 1b. Three strong diffraction peaks of metallic Cu (PDF No. 004-0836) are observed at 43.3°, 50.4°, and 74.1°. The characteristic diffraction peaks of CuO (PDF No. 045-0937) are also observed at 35.5°and 38.7° indexed to the (002) and (111) planes, respectively. Furthermore, the XRD image of CF is also exhibited in Figure S2. Additionally, the Raman spectra of CuO/CF and CuO@CoFe-LDH/CF (Figure S3) at 277, 324, and 609 cm^−1^ are assigned to the A_g_, B_g_, and B_g_ modes of CuO species, respectively [29]. The Raman peaks of CuO@CoFe-LDH/CF at 459 and 660 cm^−1^ are identified as OH-O and δ-FeOOH, respectively [30,31]. The scanning electron microscopy (SEM) images of CuO@CoFe-LDH/CF (Figure 1c,d) indicate that the CuO nanowire (Figure S4) supported on CF (Figure S5) is fully covered with a cross-linked CoFe-LDH nanosheet. The SEM and corresponding energy-dispersive X-ray (EDX) elemental mapping images of CuO@CoFe-LDH/CF further prove the existence of Co, Fe, Cu, and O elements with a homogeneous distribution (Figure 1e and Figure S6). Moreover, transmission electron microscopy (TEM) reveals the typical hierarchical structure of CuO@CoFe-LDH (Figure 1f). A high-resolution TEM (HRTEM) image of CuO@CoFe-LDH (Figure 1g) exhibits that a lattice spacing of 0.253 nm is well-indexed to the (002) plane of CuO.

**Figure 2:**
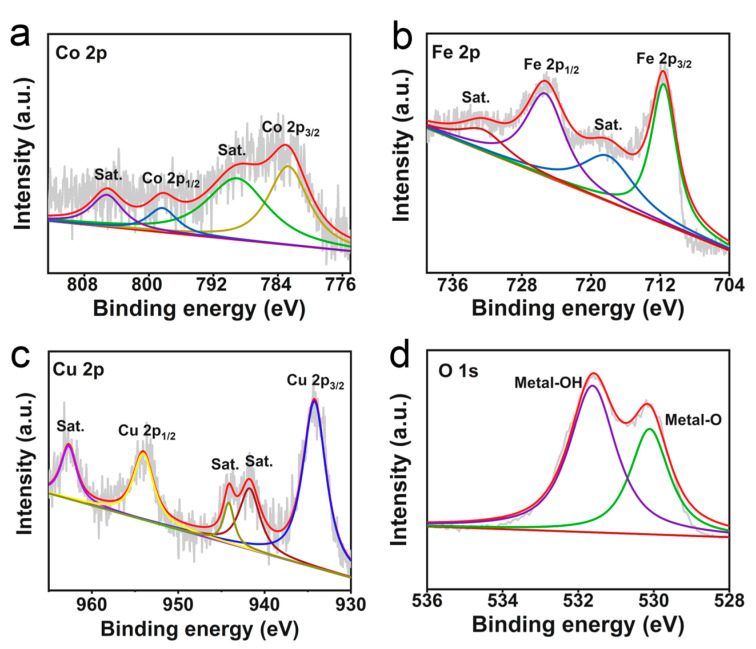
High-resolution XPS spectra of CuO@CoFe-LDH/CF in the (**a**) Co 2p, (**b**) Fe 2p, (**c**) Cu 2p, and (**d**) O 1s regions.

X-ray photoemission spectroscopy (XPS) survey spectrum (Figure S7) further demonstrates the existence of Co, Fe, Cu, and O. The Co 2p spectrum of CuO@CoFe-LDH/CF (Figure 2a) displays two dominant peaks at 782.5 and 798.3 eV for Co 2p_3/2_ and Co 2p_1/2_ of Co^2+^, respectively [32]. The two additional satellite peaks at 789.1 and 805.2 eV are attributed to Co 2p_3/2_ and Co 2p_1/2_, respectively. The Fe 2p region (Figure 2b) shows two peaks at 711.6 and 725.3 eV matched with Fe^3+^ 2p_3/2_ and Fe^3+^ 2p_1/2_ [33], respectively. In the Cu 2p spectrum of CuO@CoFe-LDH/CF, two peaks at 934.2 and 954 eV can attribute to Cu 2p_3/2_ and Cu 2p_1/2_, respectively, further affirming the presence of Cu^2+^ oxidation state (Figure 2c) [34,35]. In addition, two characteristic peaks in O 1s region (Figure 2d) at 530.1 and 531.6 eV are assigned to metal-O and metal-OH, respectively [36,37].

**Figure 3:**
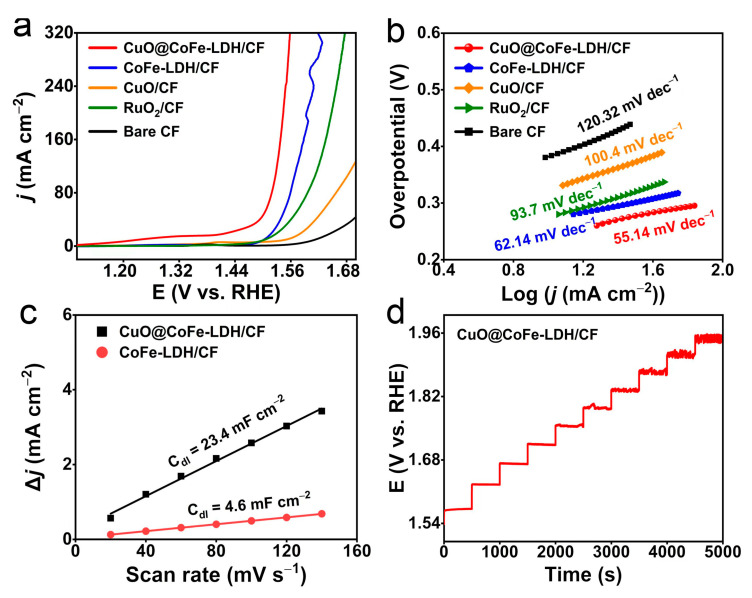
(**a**) LSV curves with the scan rate of 5 mV s^−1^ and (**b**) corresponding Tafel plots of CuO@CoFe-LDH/CF, CoFe-LDH/CF, CuO/CF, RuO_2_/CF, and CF in 1 M KOH. (**c**) Capacitive current densities at 0.975 V vs. RHE as a function of scan rate for CuO@CoFe-LDH/CF and CoFe-LDH/CF. (**d**) Chronopotentiometric test of CuO@CoFe-LDH/CF at multiple current densities steps from 20 to 200 mA cm^−2^ without iR correction.

The electrocatalytic OER performances of different working electrodes were initially investigated in 1 M KOH. The relevant linear sweep voltammetry (LSV) curves with iR-correction of the CuO@CoFe-LDH/CF, CoFe-LDH/CF, CuO/CF, RuO_2_/CF, and CF are presented in Figure 3a. Impressively, the required overpotentials at *j* of 100 and 300 mA cm^−2^ for CuO@CoFe-LDH/CF are 295 and 326 mV, respectively, which are superior to CoFe-LDH/CF (336 and 395 mV), CuO/CF (446 and 617 mV), and RuO_2_/CF (379 and 442 mV). In addition, the Tafel slope shows a key criterion of kinetic properties. As illustrated in Figure 3b, CuO@CoFe-LDH/CF attains the smallest Tafel slope of 55.14 mV dec^−1^ compared with CoFe-LDH/CF (62.14 mV dec^−1^), CuO/CF (100.4 mV dec^−1^), RuO_2_ (93.7 mV dec^−1^), and CF (120.32 mV dec^−1^), reflecting CuO@CoFe-LDH/CF has the fastest OER reaction kinetics. Notably, the double-layer capacitance (C_dl_) value is evaluated by cyclic voltammetry tests in the non-Faraday region (Figure S8) of CuO@CoFe-LDH/CF is 5.08 times as large as the CoFe-LDH/CF (23.4 vs. 4.6 mF·cm^−2^) (Figure 3c), signifying CuO@CoFe-LDH/CF can expose abundant active sites. The multi-step chronopotentiometry curve (Figure 3d) shows that the potentials are rapidly stabilized at each step, indicating CuO@CoFe-LDH/CF has a remarkable mass transfer capability.

**Figure 4:**
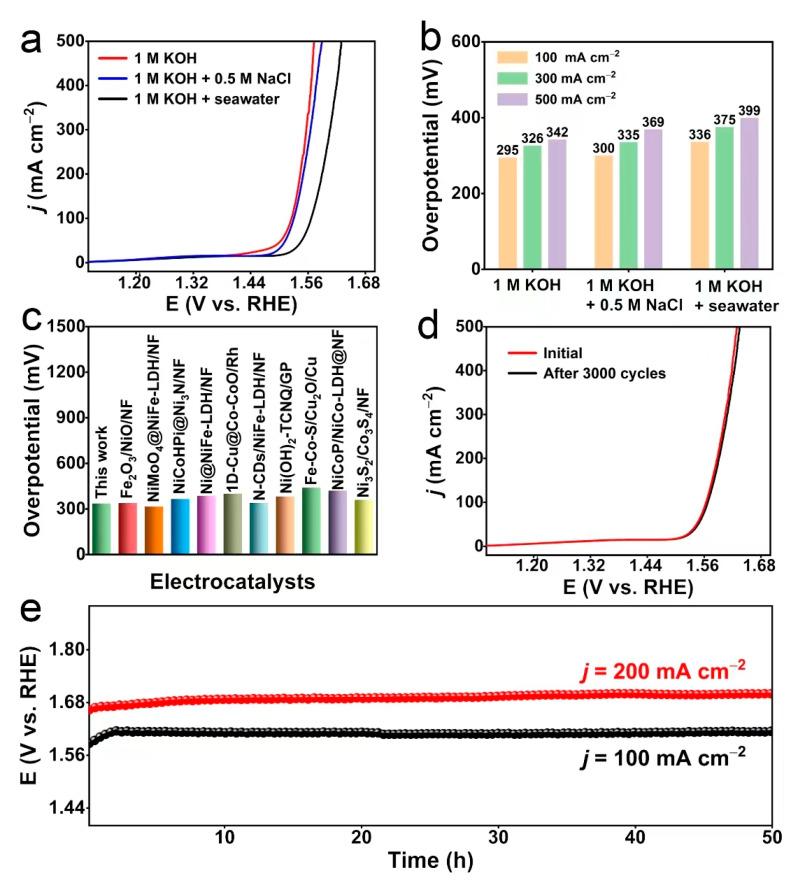
(**a**) LSV curves and (**b**) corresponding overpotentials of the CuO@CoFe-LDH/CF in different electrolytes. (**c**) Comparison of overpotentials at 100 mA cm^−2^ between the CuO@CoFe-LDH/CF and other reported self-supported seawater OER catalysts. (**d**) LSV curves of CuO@CoFe-LDH/CF with the scan rate of 5 mV s^−1^ before and after 3000 CV cycles from 1.4 to 1.7 V vs. RHE. (**e**) Chronopotentiometry curves of CuO@CoFe-LDH/CF at 100 and 200 mA cm^−2^ in M KOH + seawater without iR correction.

Motivated by the superior OER catalytic performance of CuO@CoFe-LDH/CF in alkaline freshwater, it was further evaluated in alkaline simulated seawater and alkaline seawater. When measured in alkaline seawater (black curve), the catalytic activity of CuO@CoFe-LDH/CF is less desirable than that in 1 M KOH (red curve) and alkaline simulated seawater (blue curve) in Figure 4a, which may result from the complex composition of seawater. Noticeably, the needed overpotentials of CuO@CoFe-LDH/CF to achieve *j* of 100, 300, and 500 mA cm^−2^ are only 336, 375, and 399 mV, respectively, revealing CuO@CoFe-LDH/CF has excellent seawater oxidation activity (Figure 4b). As displayed in Figure S9, CuO@CoFe-LDH/CF shows a Tafel slope of 55.14 mV dec^−1^, 56.62 mV dec^−1^, and 70.2 mV dec^−1^ in alkaline freshwater, alkaline simulated seawater, and alkaline seawater, respectively. Notably, the electrocatalytic performance of CuO@CoFe-LDH/CF to generate the *j* of 100 mA cm^−2^ also stands out from most of the reported OER self-supported seawater electrocatalysts (Figure 4c and Table S1). Figure 4d exhibits the LSV curves of CuO@CoFe-LDH/CF before (red curve) and after 3000 CV scans (black curve), and it shows no noticeable decay in comparison to the initial one before scanning. Furthermore, the chronopotentiometry tests conducted at *j* of 100 and 200 mA cm^−2^ are also applied to show remarkable OER stability of the CuO@CoFe-LDH/CF in alkaline seawater, which shows no significant decay after 50 h operation (Figure 4e). In contrast, CoFe-LDH/CF exhibits obvious performance degradation after only 24 h of continuous electrolysis (Figure S10). Colorimetric test papers are used to confirm the existence of hypochlorite production during seawater oxidation. Figure S11 shows no apparent color change in the test papers, indicating that hypochlorite is not produced in the stability tests. The SEM images (Figure S12) and XRD pattern (Figure S13) of the post-OER CuO@CoFe-LDH/CF confirm that the morphology and crystal structure of CuO@CoFe-LDH/CF are almost unchanged, suggesting the excellent stability of CuO@CoFe-LDH/CF in alkaline seawater. Notably, no peak associated with Cl is observed in the XPS survey spectrum of post-OER CuO@CoFe-LDH/CF (Figure S14a). Moreover, the cobalt in CuO@CoFe-LDH/CF is oxidized to higher valance Co^3+^ (CoOOH) after a long-term durability test (Figure S14b), which may be the active site for OER. The production of CoOOH is useful for the resistance to chloride ion corrosion in seawater [26].

## 3. Materials and Methods

### 3.1. Materials

Hydrochloric acid (HCl), Ethanol (C_2_H_5_OH), Sodium chloride (NaCl), Iron(Ⅱ) sulfate heptahydrate (FeSO_4_·7H_2_O), Sodium hydroxide (NaOH), Ammonium persulfate [(NH_4_)_2_S_2_O_8_], Cobalt(Ⅱ) nitrate hexahydrate (Co(NO_3_)_2_·6H_2_O), Ruthenium oxide (RuO_2_), and Nafion (5 wt%) were obtained from Aladdin Industrial Co. Ltd. (Shanghai, China), Sodium carbonate (Na_2_CO_3_), potassium hydroxide (KOH) were obtained from Chengdu Kelong Chemical Reagent Factory (Chengdu, China). Cu foam (CF) was purchased from Shenzhen Green and Creative Environmental Science and Technology Co. Ltd. (Shenzhen, China), Natural seawater was collected from Weihai, Shandong, China, and most of the magnesium and calcium salts were removed by first adding 3.4 g Na_2_CO_3_ to 500 mL of natural seawater before use.

### 3.2. Preparation of CuO/CF, CuO@CoFe-LDH/CF, and CoFe-LDH/CF

Firstly, 5 g NaOH and 1.428 g (NH_4_)_2_S_2_O_8_ were dissolved in 50 mL of ultrapure water and then put a Cu foam (2 cm × 3 cm) into the aqueous solution for 20 min. The obtained sample was dried in the air, followed by air annealing at 180 °C for 1 h (2 °C min^−1^) to obtain CuO/CF. After that, potentiostatic electrodeposition was performed in a three-electrode setup. The working electrode, reference electrode, and counter electrode were the CuO/CF, Ag/AgCl, and a graphite rod, separately. Typically, Co(NO_3_)_2_·6H_2_O (2.2 g) and FeSO_4_·7H_2_O (2.08 g) were dissolved in 50 mL of water and mixed to form the electrolyte. Then, the CoFe-LDH was electrodeposited on the CuO (1 cm × 2 cm) at −1.0 V vs. Ag/AgCl for 100 s. The synthesized catalyst was washed with water several times and dried in air. CoFe-LDH/CF was similarly prepared.

### 3.3. Characterizations

X-ray diffraction (XRD) was tested using a LabX XRD-6100 X-ray diffractometer (SHIMADZU, Kyoto, Japan). Scanning electron microscopy (SEM) images were obtained via a GeminiSEM 300 microscope (ZEISS, Oberkochen, Germany). Transmission electron microscopy (TEM) images were acquired on JEM-F200 Multi-purpose Electron Microscope (JEOL, Tokyo, Japan). X-ray photoelectron spectroscopy (XPS) was conducted using ESCALABMK II X-ray photoelectron spectrometer (Thermo, Waltham, MA, America). In situ Raman spectroscopy was recorded on the Horiba-Xplora Plus confocal microscope with 633 nm (HORIBA, Kyoto, Japan).

## 4. Conclusions

In summary, we report a hierarchical CuO@CoFe-LDH nanoarray on Cu foam as a high-active and robust seawater oxidation electrocatalyst. Such CuO@CoFe-LDH/CF offers excellent electrocatalytic activity for seawater oxidation with low overpotentials of only 336 and 375 mV to attain *j* of 100 and 300 mA cm^−2^, respectively. It also shows long-term electrochemical durability to retain its activity for at least 50 h at a *j* of 200 mA cm^−2^. This work not only offers an efficient and stable catalyst for seawater oxidation but also paves the strategy for the construction of core–shell hierarchical nanoarray as attractive catalyst materials for seawater oxidation.

## Data Availability

Not applicable.

## Supplementary Materials

The following supporting information can be downloaded at: https://www.mdpi.com/article/10.3390/molecules28155718/s1. Figure S1: Optical photograph of CF, Cu(OH)_2_/CF, CuO/CF, and CuO@CoFe-LDH/CF; Figure S2: XRD pattern of bare CF; Figure S3: Raman spectra of CuO/CF and CuO@CoFe-LDH/CF; Figure S4: (a) Low- and (b) high-magnification SEM images of CuO/CF; Figure S5: (a) Low- and (b) high-magnification SEM images of bare CF; Figure S6: EDX spectrum of CuO@CoFe-LDH/CF; Figure S7: XPS survey spectrum of CuO@CoFe-LDH/CF; Figure S8: CV curves for (a) CuO@CoFe-LDH/CF and (b) CoFe-LDH/CF in the double layer region at different scan rates of 20, 40, 60, 80, 100, 120, and 140 mV s^−1^ in 1 M KOH electrolyte; Figure S9: Tafel plots for CuO@CoFe-LDH/CF in 1 M KOH, 1 M KOH + 0.5 M NaCl, and 1 M KOH + seawater electrolyte; Figure S10: Chronopotentiometry curve of CoFe-LDH/CF at 100 mA cm^−2^ in 1 M KOH + seawater electrolyte; Figure S11: Optical photograph of the colorimetric paper testing result of hypochlorite production in 1 M KOH + seawater electrolyte before and after durability test of CuO@CoFe-LDH/CF at current densities of 100 and 200 mA cm^−2^; Figure S12: (a) Low- and (b) high-magnification SEM images of post-OER CuO@CoFe-LDH/CF in 1 M KOH + seawater electrolyte; Figure S13: XRD pattern of post-OER CuO@CoFe-LDH/CF in 1 M KOH + seawater electrolyte; Figure S14: (a) XPS survey spectrum of post-OER CuO@CoFe-LDH/CF in 1 M KOH + seawater electrolyte. High-resolution XPS spectra for post-OER CuO@CoFe-LDH/CF tested in 1 M KOH + seawater electrolyte in the (b) Co 2p, (c) Fe 2p, (d) Cu 2p, and (e) O 1s regions; Table S1: Comparison of OER performances for CuO@CoFe-LDH/CF with other reported self-supported electrocatalysts [38,39,40,41,42,43,44,45,46,47,48].
