# Supplementary material for: A Hierarchical CuO Nanowire@CoFe-Layered Double Hydroxide Nanosheet Array as a High-Efficiency Seawater Oxidation Electrocatalyst
Source: Molecules. 2023 Jul 28;28(15):5718. doi: 10.3390/molecules28155718 (PMC10420605; doi:10.3390/molecules28155718)
Supplement: Supplementary file 1 [file molecules-28-05718-s001.zip › molecules-2447906-supplementary.pdf]

Supplementary Material

A hierarchical CuO nanowire@CoFe-layered double hydroxide nanosheet array as a high-efficiency seawater oxidation electrocatalyst

Electrochemical tests:

Electrochemical OER experiments were performed with the CHI 760E electrochemical workstation, using the prepared samples (1 × 0.5 cm^2), carbon rod, and Hg/HgO electrode as the working electrode, counter electrode, and reference electrode, respectively. Three different electrolytes, including 1 M KOH, 1 M KOH + 0.5 M NaCl, and 1 M KOH + seawater, were used, and the pH was about 14.0. All measured potentials were referenced to that of reversible hydrogen electrode (RHE) ($E_{RHE} = E_{Hg/HgO} + 0.059 \times pH + 0.098$ V). The catalytic activity of catalysts was determined by linear sweep voltammetry (LSV) curves with a scan rate of 5 mV s^{-1}. The double-layer capacitance (C_{dl}) values were obtained via cyclic voltammetry (CV) curves with the scan rates of 20−140 mV s^{-1}. All data (except for Figure 3d, Figure 4e, and Figure S10) have been reported with iR compensation. The iR-compensated potential was obtained after the correction of solution resistance measured following the equation: Ecorr = E − iR, where E is the original potential, R is the solution resistance, i is the corresponding current, and Ecorr is the iR-compensated potential.

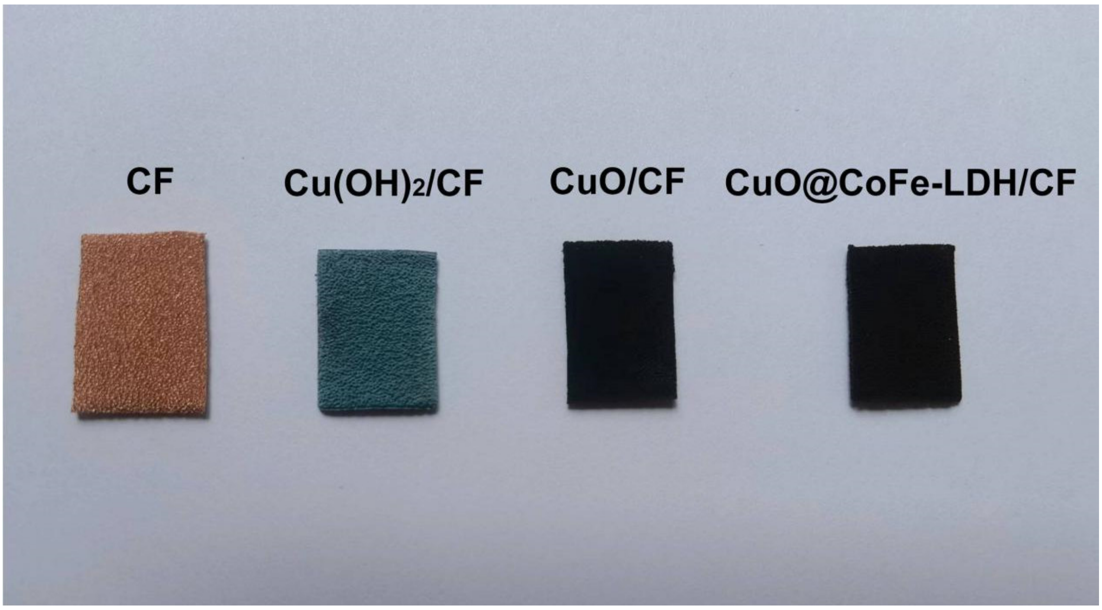

Figure S1. Optical photograph of CF, $Cu(OH)_2$/CF, CuO/CF, and CuO@CoFe-LDH/CF.

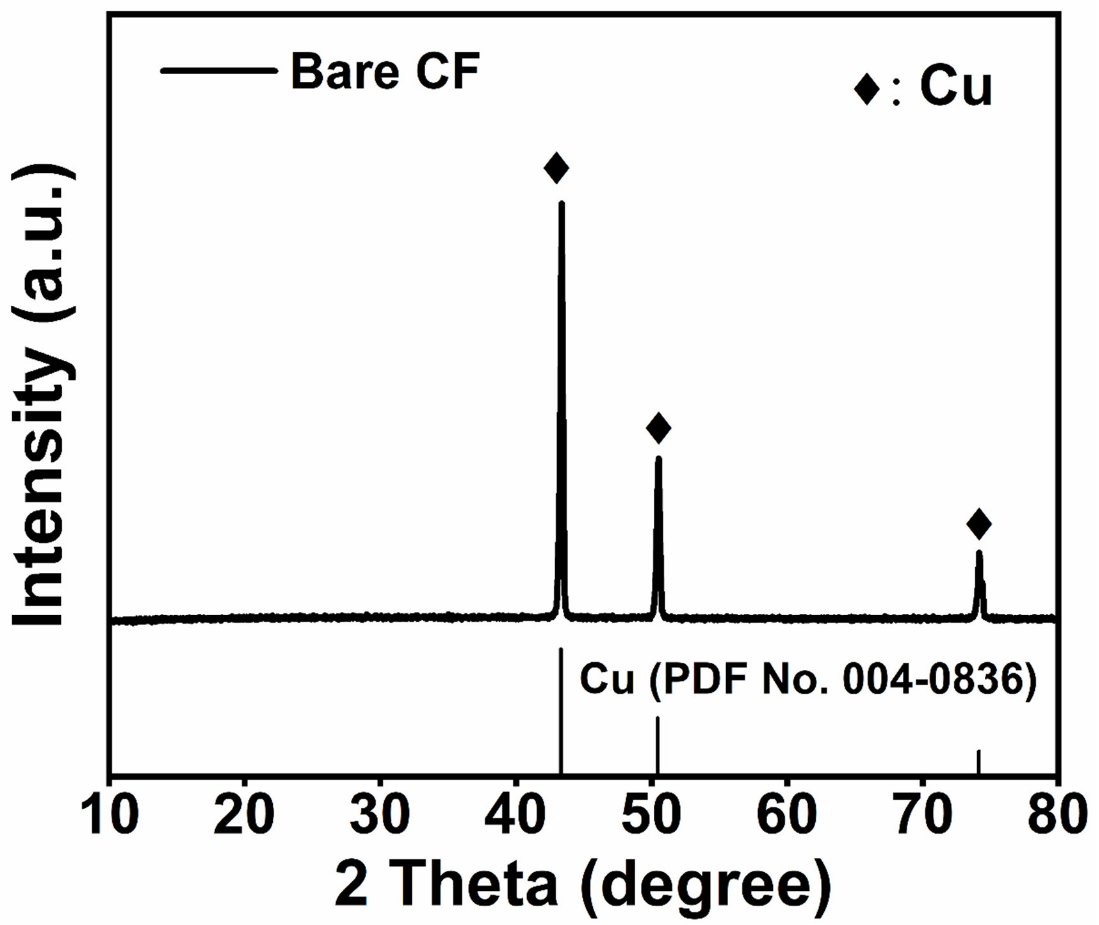

Figure S2. XRD pattern of bare CF.

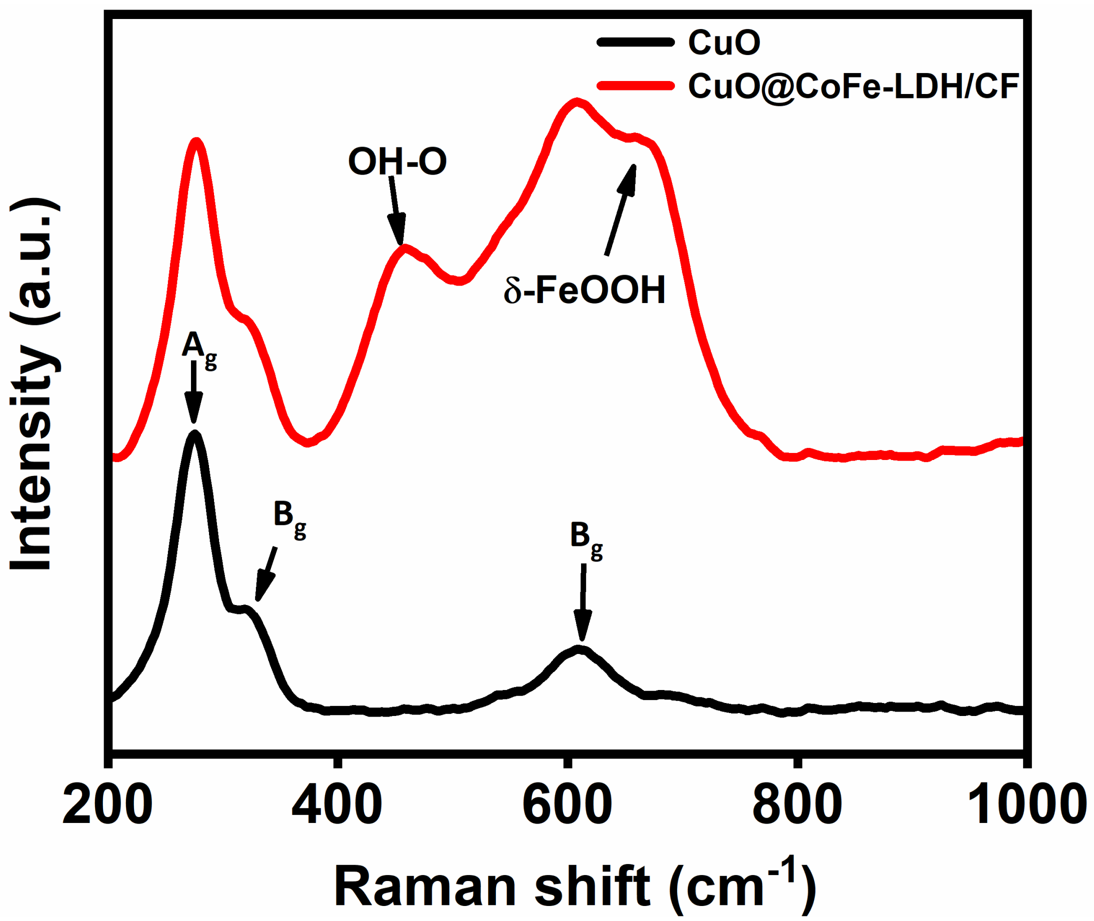

Figure S3. Raman spectra of CuO/CF and CuO@CoFe-LDH/CF.

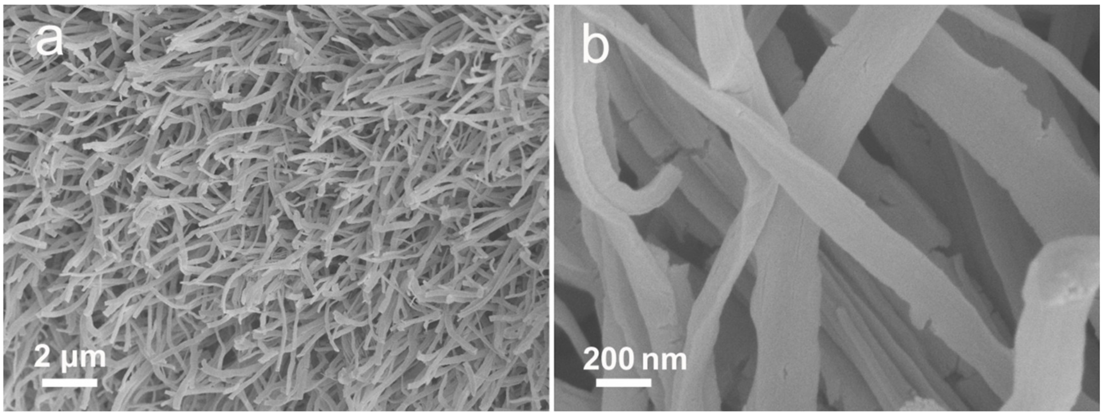

Figure S4. (a) Low- and (b) high-magnification SEM images of CuO/CF.

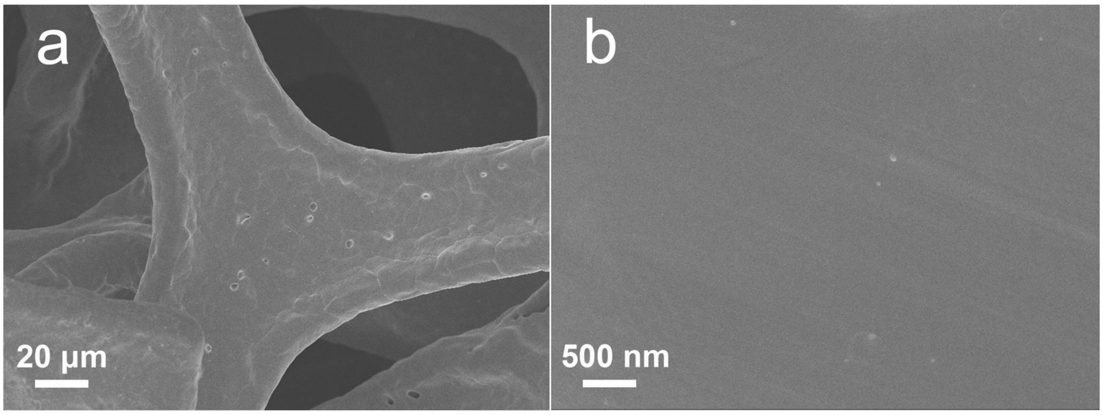

Figure S5. (a) Low- and (b) high-magnification SEM images of bare CF.

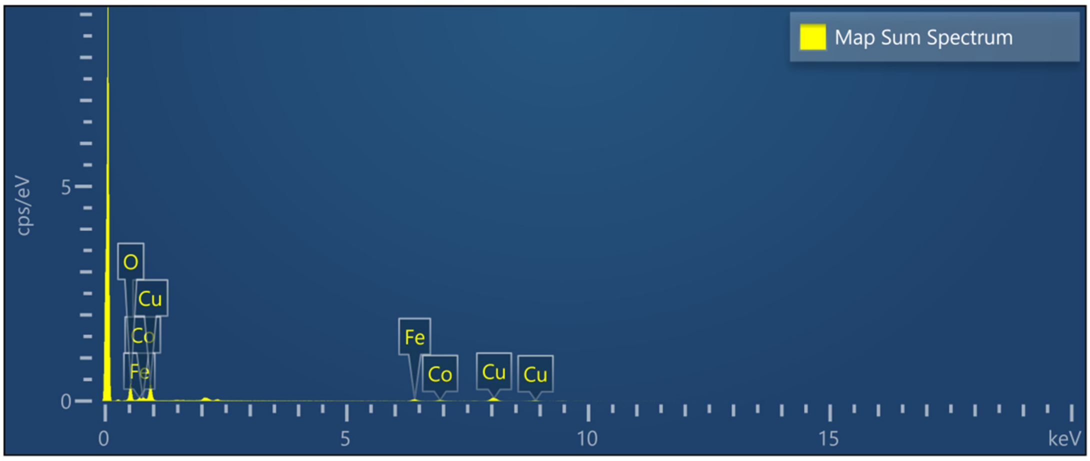

Figure S6. EDX spectrum of CuO@CoFe-LDH/CF.

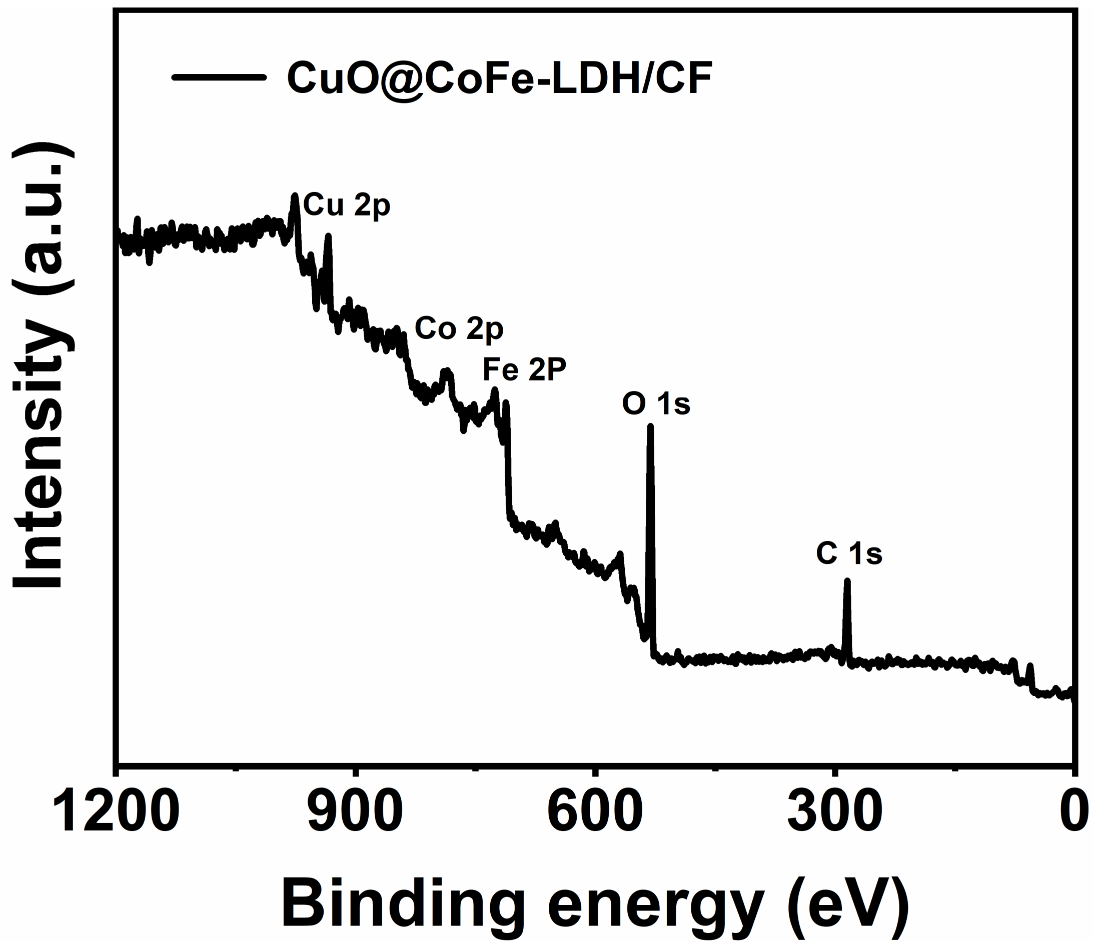

Figure S7. XPS survey spectrum of CuO@CoFe-LDH/CF.

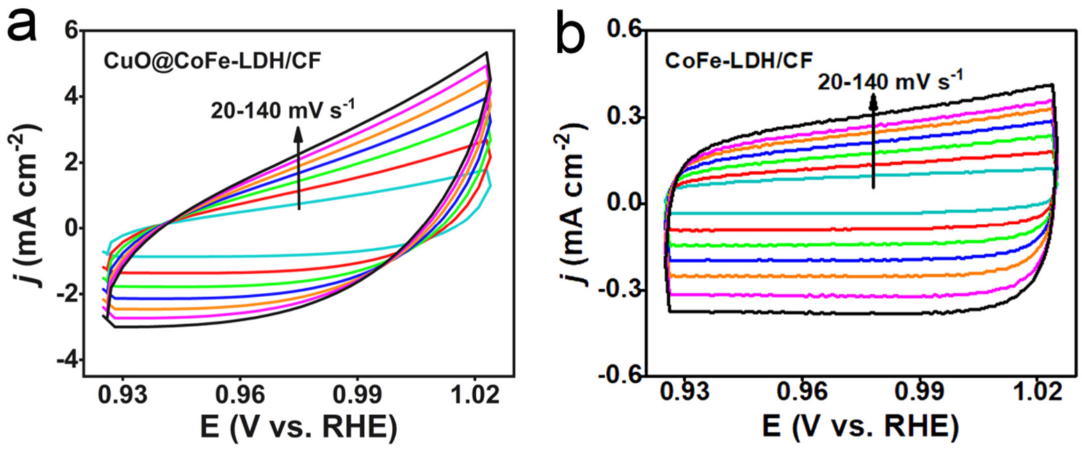

Figure S8. CV curves for (a) CuO@CoFe-LDH/CF and (b) CoFe-LDH/CF in the double layer region at different scan rates of 20, 40, 60, 80, 100, 120, and 140 mV s^{-1} in 1 M KOH electrolyte.

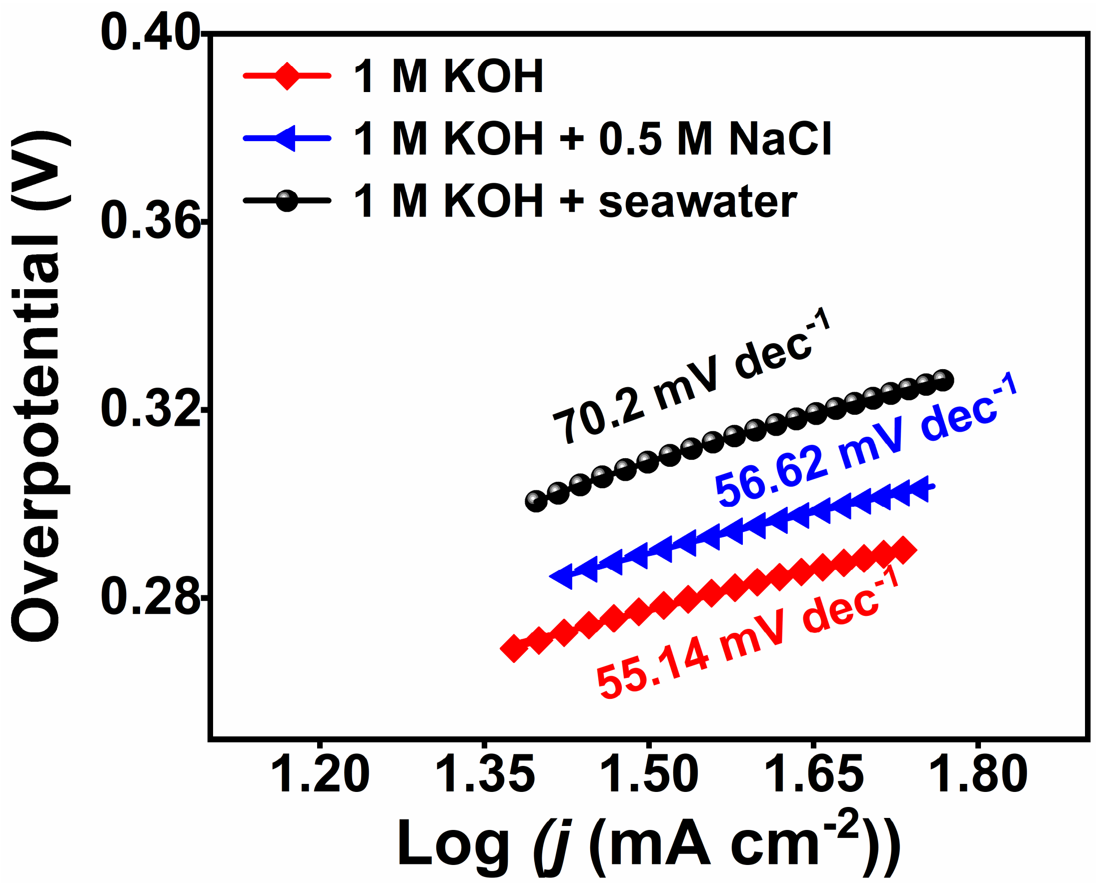

Figure S9. Tafel plots for CuO@CoFe-LDH/CF in 1 M KOH, 1 M KOH + 0.5 M NaCl, and 1 M KOH + seawater electrolyte.

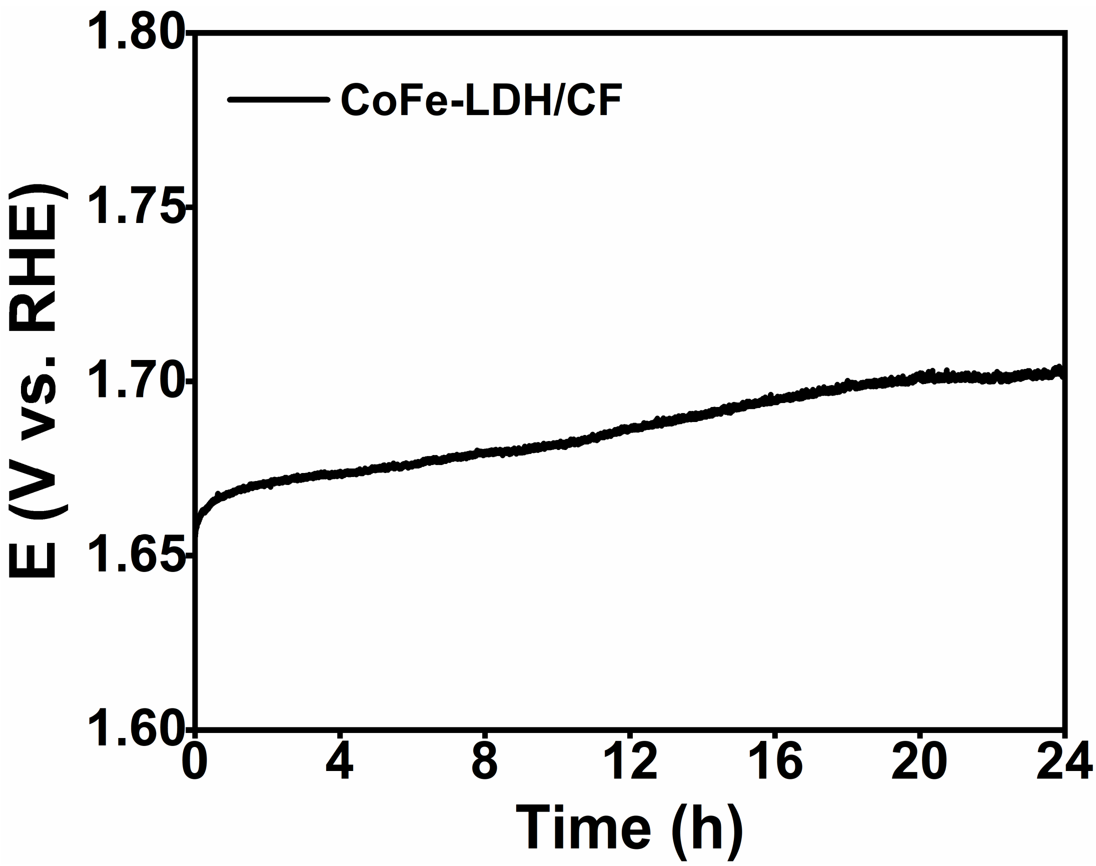

Figure S10. Chronopotentiometry curve of CoFe-LDH/CF at 100 mA cm^{-2} in 1 M KOH + seawater electrolyte.

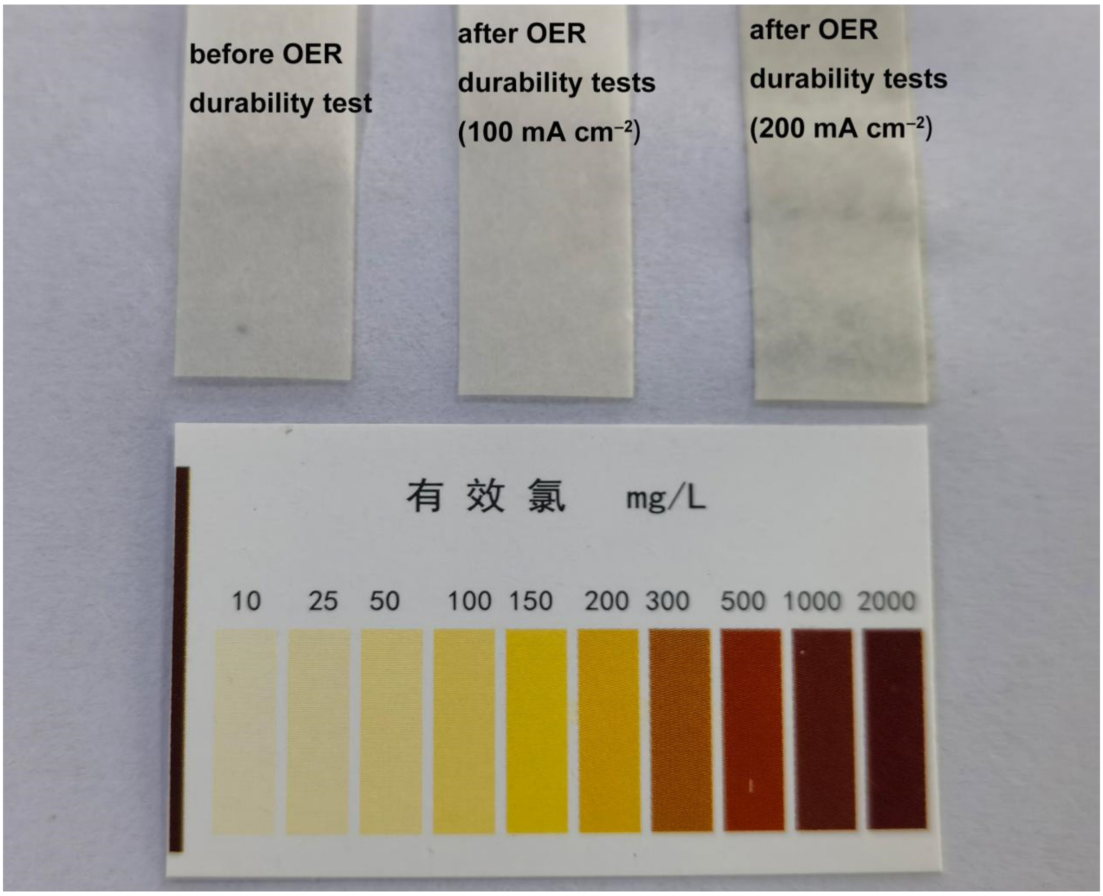

Figure S11. Optical photograph of the colorimetric paper testing result of hypochlorite production in 1 M KOH + seawater electrolyte before and after durability test of CuO@CoFe-LDH/CF at current densities of 100 and 200 mA cm^{-2}.

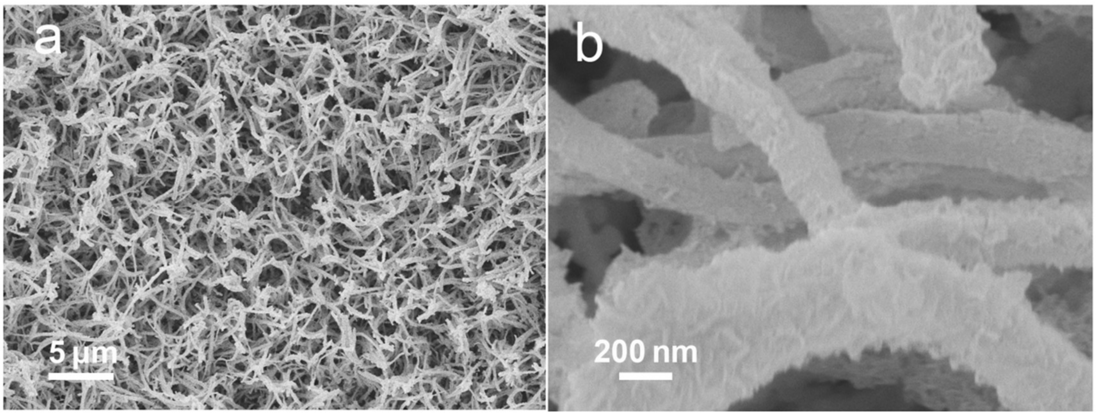

Figure S12. (a) Low- and (b) high-magnification SEM images of post-OER CuO@CoFe-LDH/CF in 1 M KOH + seawater electrolyte.

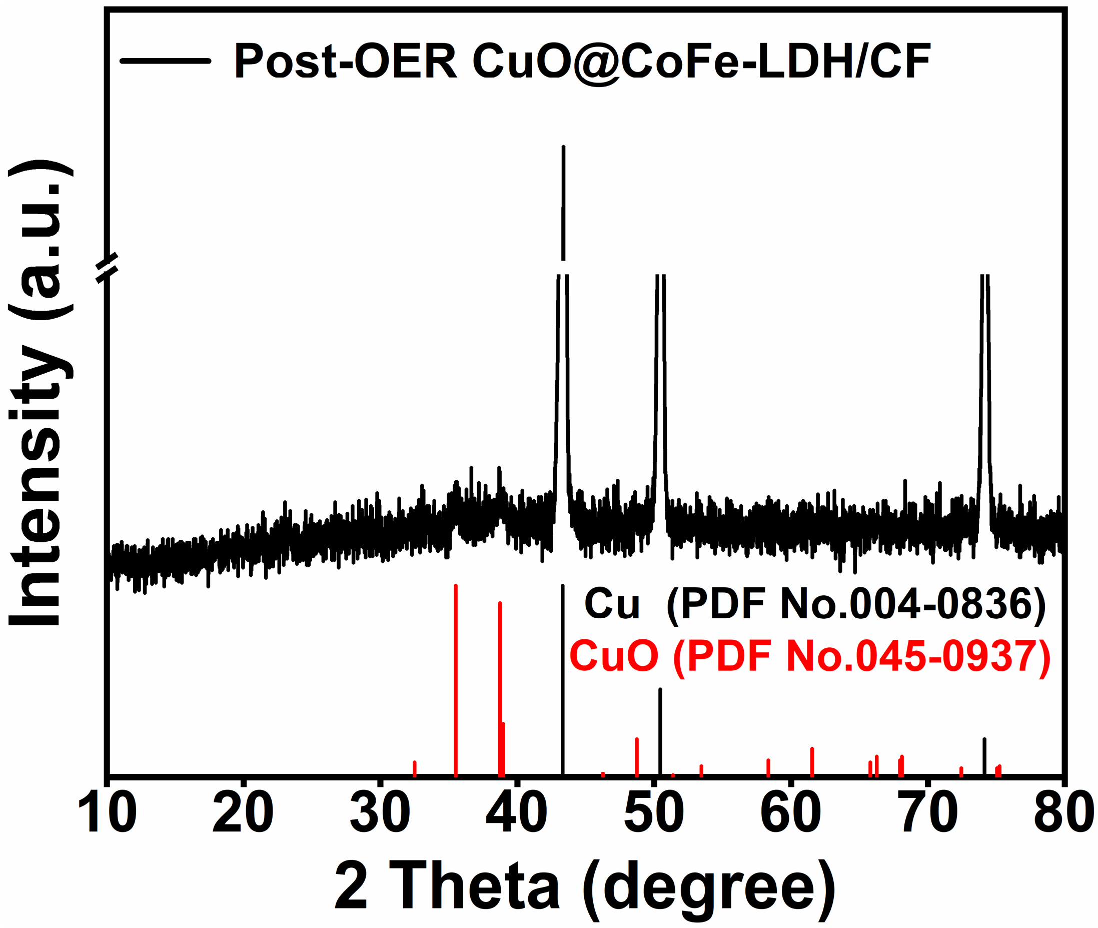

Figure S13. XRD pattern of post-OER CuO@CoFe-LDH/CF in 1 M KOH + seawater electrolyte.

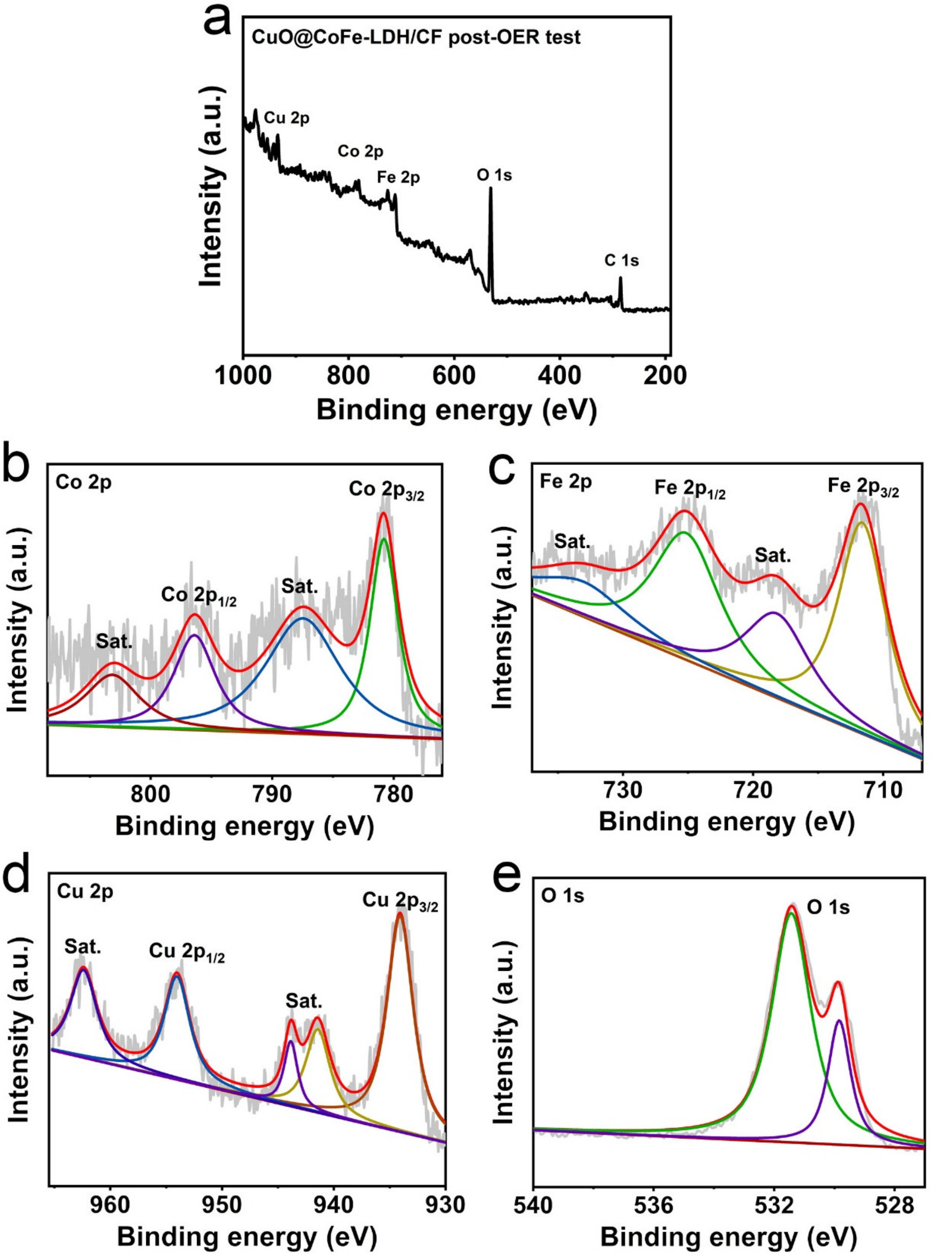

Figure S14. (a) XPS survey spectrum of post-OER CuO@CoFe-LDH/CF in 1 M KOH + seawater electrolyte. High-resolution XPS spectra for post-OER CuO@CoFe-LDH/CF tested in 1 M KOH + seawater electrolyte in the (b) Co 2p, (c) Fe 2p, (d) Cu 2p, and (e) O 1s regions.

Table S1. Comparison of OER performances for CuO@CoFe-LDH/CF with other reported self-supported electrocatalysts.

Catalyst	Electrolyte	η_{100}*(mV)	Ref.
CuO@CoFe-LDH/CF	1 M KOH	302	This work
	1 M KOH + seawater	336	
Fe_2O_3/NiO/NF	1 M KOH	324	[1]
	1 M KOH + seawater	339	
S-$NiMoO_4$@NiFe-LDH/NF	1 M KOH	273	[2]
	1 M KOH + seawater	315	
NiCoHPi@Ni_3N/NF	1 M KOH	342	[3]
	1 M KOH + seawater	365	
Ni@NiFe-LDH/NF	1 M KOH	312	[4]
	1 M KOH + seawater	386	
1D-Cu@Co-CoO/Rh	1 M KOH	380	[5]
	1 M KOH + seawater	400	
N-CDs/NiFe-LDH/NF	1 M KOH	260	[6]
	1 M KOH + seawater	340	
$Ni(OH)_2$-TCNQ/GP	1 M KOH	340	[7]
	1 M KOH + seawater	382	
Fe-Co-S/Cu_2O/Cu	1 M KOH	390	[8]
	1 M KOH + seawater	440	
NiMoN@NiFeN/NF	1 M KOH	277	[9]
	1 M KOH + seawater	307	
Ni_3S_2/Co_3S_4/NF	1 M KOH	270	[10]
	1 M KOH + seawater	360	
NiCoP/NiCo-LDH@NF	1 M KOH	370	[11]
	1 M KOH + seawater	420	
Pt-CoFe(II)-LDHs/NF	1 M KOH	272	[12]
	1 M KOH + seawater	302	
CuB_x@PU	1 M KOH	/	[13]
	1 M KOH + 0.5 M NaCl	416	

η_{100}* represent the overpotentials required to attain j of 100 mA cm^{-2}.

References

1 Li, L.; Zhang, G.; Wang, B.; Zhu, D.; Liu, D.; Liu, Y.; Yang, S. Fe_2O_3/NiO Interface for the electrochemical oxygen evolution in seawater and domestic sewage. *ACS Appl. Mater. Interfaces* **2021**, *13*, 37152–37161.

2 Wang, H.; Chen, L.; Tan, L.; Liu, X.; Wen, Y.; Hou, W.; Zhan, T. Electrodeposition of NiFe-layered double hydroxide layer on sulfur-modified nickel molybdate nanorods for highly efficient seawater splitting. *J. Colloid Interface Sci.* **2022**, *613*, 349–358.

3 Sun, H.; Sun, J.; Song, Y.; Zhang, Y.; Qiu, Y.; Sun, M.; Tian, X.; Li, C.; Lv, Z.; Zhang, L. Nickel–Cobalt hydrogen phosphate on nickel nitride supported on nickel foam for alkaline seawater electrolysis. *ACS Appl. Mater. Interfaces* **2022**, *14*, 22061–22070.

4 Zhang, F.; Liu, Y.; Wu, L.; Ning, M.; Song, S.; Xiao, X.; Hadjiev, V. G.; Fan, D. E.; Wang, D.; Yu, L.; Chen, S.; Ren, Z. Efficient alkaline seawater oxidation by a three-dimensional core-shell dendritic NiCo@NiFe layered double hydroxide electrode. *Mater. Today Phys.* **2022**, *27*, 100841.

5 Tran, P. K. L.; Tran, D. T.; Malhotra, D.; Prabhakaran, S.; Kim, D. H.; Kim, N. H.; Lee, J. H. Highly effective freshwater and seawater eelectrolysis enabled by atomic rh-modulated Co-CoO lateral heterostructures. *Small* **2021**, *17*, 2103826.

6 Ding, P.; Song, H.; Chang, J.; Lu, S. N-doped carbon dots coupled NiFe-LDH hybrids for robust electrocatalytic alkaline water and seawater oxidation. *Nano Res.* **2022**, *15*, 7063–7070.

7 Zhang, L.; Wang, J.; Liu, P.; Liang, J.; Luo, Y.; Cui, G.; Tang, B.; Liu, Q.; Yan, X.; Hao, H.; Liu, M.; Gao, R.; Sun, X. $Ni(OH)_2$ nanoparticles encapsulated in conductive nanowire array for high-performance alkaline seawater oxidation. *Nano Res.* **2022**, *15*, 6084–6090.

8 Sun, J.; Song, P.; Zhou, H.; Lang, L.; Shen, X.; Liu, Y.; Cheng, X.; Fu, X.; Zhu, G. A surface configuration strategy to hierarchical Fe-Co-S/Cu_2O/Cu electrodes for oxygen evolution in water/seawater splitting. *Appl. Surf. Sci.* **2021**, *567*, 150757.

9 Yu, L.; Zhu, Q.; Song, S.; McElhenny, B.; Wang, D.; Wu, C.; Qin, Z.; Bao, J.; Yu, Y.; Chen, S.; Ren, Z. Non-noble metal-nitride based electrocatalysts for high-performance alkaline seawater electrolysis. *Nat. Commun.* **2019**, *10*, 5106.

10 Wang, C.; Zhu, M.; Cao, Z.; Zhu, P.; Cao, Y.; Xu, X.; Xu, C.; Yin, Z. Heterogeneous bimetallic sulfides based seawater electrolysis towards stable industrial-level large current density. *Appl. Catal. B* **2021**, *291*, 120071.

11 Wu, Y.; Tian, Z.; Yuan, S.; Qi, Z.; Feng, Y.; Wang, Y.; Huang, R.; Zhao, Y.; Sun, J.; Zhao, W.; Guo, W.; Feng, J.; Sun, J. Solar-driven self-powered alkaline seawater electrolysis via multifunctional earth-abundant heterostructures. *Chem. Eng. J.* **2021**, *411*, 128538.

12 Wu, J.; Nie, Z.; Xie, R.; Hu, X.; Yu, Y.; Yang, N. Self-assembled Pt–CoFe layered double hydroxides for efficient alkaline water/seawater splitting by spontaneous redox synthesis. *J. Power Sources* **2022**, *532*, 231353.

13 Zhang, Y.; Fu, C.; Weng, S.; Lv, H.; Li, P.; Deng, S.; Hao, W. Construction of an "environment-friendly" CuB_x@PU self-supporting electrode toward efficient seawater electrolysis. *Green Chem.* **2022**, *24*, 5918–5929.
